# Hydrogen bonding-induced high-performance stretchable organic semiconductors: a Review

**DOI:** 10.3389/fchem.2023.1200644

**Published:** 2023-04-21

**Authors:** Jinhan Chen, Zheng Wang, Zhifeng Deng, Ligui Chen, Xuhui Wu, Yihan Gao, Yumeng Hu, Mei Li, Hongzhen Wang

**Affiliations:** ^1^ National and Local Joint Engineering Laboratory for Slag Comprehensive Utilization and Environmental Technology, School of Materials Science and Engineering, Shaanxi University of Technology (SNUT), Hanzhong, Shaanxi, China; ^2^ Key Laboratory of Rubber–Plastic of Ministry of Education (QUST), School of Polymer Science and Engineering, Qingdao University of Science and Technology, Qingdao, China

**Keywords:** hydrogen bonding, stretchable organic semiconductors, charge mobility, softelectron devices, OSCs, OFETs

## Abstract

Semiconductors are widely used in electron devices. With the development of wearable soft-electron devices, conventional inorganic semiconductors are unable to meet the demand because of their high rigidity and high cost. Thus, scientists construct organic semiconductors with high charge mobility, low cost, eco-friendly, stretchable, etc. Due to the excellent performance of stretchable organic semiconductors, they can be widely used as wearable soft-electron devices, such as stretchable organic field-effect transistors (OFETs), organic solar cells (OSCs), etc. Contains flexible display devices and flexible power sources, which are of great interest for applications of future electron devices. However, there are still some challenges that need to be solved. Commonly, enhancing the stretchability may cause the degradation of charge mobility, because of the destruction of the conjugated system. Currently, scientists find that hydrogen bonding can enhance the stretchability of organic semiconductors with high charge mobility. Thus in this review, based on the structure and design strategies of hydrogen bonding, various hydrogen bonding induced stretchable organic semiconductors are introduced. In addition, the applications of the hydrogen bonding induced stretchable organic semiconductors are reviewed. Finally, the stretchable organic semiconductors design concept and potential evolution trends are discussed. The final goal is to outline a theoretical scaffold for the design of high-performance wearable soft-electron devices, which can also further advance the development of stretchable organic semiconductors for applications.

## 1 Introduction

Organic semiconductors are organic materials with semiconductor properties (Chen et al., 2023). Compared with inorganic semiconductors, organic semiconductors have many advantages, such as being tailorable, easy to modify, and having low energy consumption (Zheng et al., 2022). Researchers have developed many meaningful organic semiconductor molecules, such as thiophenes, azole, fullerenes, perylene, phthalocyanines, etc. (Dai et al., 2022; Miao et al., 2023) With the further development of organic semiconductor devices, people are concentrating more on the stretchability of organic semiconductors with high charge mobility (Yang J. C. et al., 2019). The stretchable organic semiconductors make the flexible display, flexible organic sensors, and stretchable organic solar cells possible, which have shown great potential for commercialization (Yu et al., 2019).

In application, stretchable organic semiconductors will experience the recycling of stretch and retract. Therefore, for practical application, stretchable organic semiconductors need to function more than just single stretchable, but rather high elasticity and reversibility (Yu et al., 2021). However, organic semiconductor materials commonly are conjugated structures with high rigidity. Enhancing the stretchability may destroy the rigid π-conjugated system, then cause the reduction of charge mobility. Thus, designing high-stretch organic semiconductors with high charge mobility becomes a challenge (Pei et al., 2022). In addition, it is crucial to maintain high charge mobility upon the stretching status. To solve these problems, scientists have developed many useful strategies, such as embedding the crystallites into the amorphous regions, introducing side-chain, etc. (Yang Y. et al., 2019) Currently, many scientists use hydrogen bonding as the non-covalent cross-linking sites to design stretchable organic semiconductors, which have great performance in application (Charron et al., 2018).

Hydrogen bonding is an interaction force formed between hydrogen atoms and strongly electronegative atoms, which is widely existing in nature, including DNA (deoxyribonucleic acid), water, amino acid, etc. (Aakeröy and Seddon, 1993) Hydrogen bonding makes these structures more stable (Arunan et al., 2011). Same as them, hydrogen bonding can significantly enhance the stability and performance of organic semiconductor devices (Wang et al., 2018b). On the one hand, hydrogen bonding can enhance the intermolecular force and reduce the distance between molecules, which results in better π-π accumulation (Zhang et al., 2022). Because of the better π-π accumulation, the carrier mobility of stretchable organic semiconductors is elevated, which also solves the charge mobility reduction by the destruction of π-conjugation. On the other hand, the hydrongen bonds between molecules can significantly improve the stretchable properties of the systems. Thus, the design strategies of conjugated systems capable of hydrogen bonding are expected to both have high charge mobility and high stretchable properties (Lee et al., 2020).

In this review, we first summarize the recent advances in hydrogen bonding stretchable organic semiconductors in practical applications. Although some articles have reviewed the stretchable materials, only a few articles have discussed the hydrogen bonding-induced stretchable organic semiconductors. Herein, different hydrogen bonding design strategies in applications such as OSCs and OFETs are reviewed. Moreover, high-stretchable materials which are potentially used for these territories are introduced. In the end, the future outlook is highlighted. The final goal is to outline a theoretical scaffold for the design of high-performance hydrogen bonding stretchable organic semiconductors that can at the same time further the development of the applications of stretchable organic semiconductors.

## 2 OSCs

Organic solar cells as a green energy technology are attracting the attention of many scientists. Compared to conventional silicon-based solar cells, OSCs have many advantages, such as light-weight, low-cost, low pollution, etc. (Chen et al., 2021) Moreover, conventional silicon-based solar cells commonly are high rigidity, which restricts the development of soft-electronic devices (Wang et al., 2021). Relatively, because of the designability and softness of the organic materials, the OSCs are potential to be the most powerful source of soft-electronic devices (Lee et al., 2022a). The active layer of OSCs can generate the exciton when illuminated by light, then the exciton will separate into a hole and an electron. The hole and the electron can transport by the corresponding hole transport layer or electron transport layer. In this way, the current can be generated in the system. In a word, the active layer is one of the most important structures in the OSCs, which directly influences the performance of the OSCs (Liu et al., 2021). However, the active layer materials commonly are high rigidity. Thus, to promote the development of stretchable OSCs, exploiting high-charge mobility active layers with high stretchability is immediate.

**FIGURE 1:**
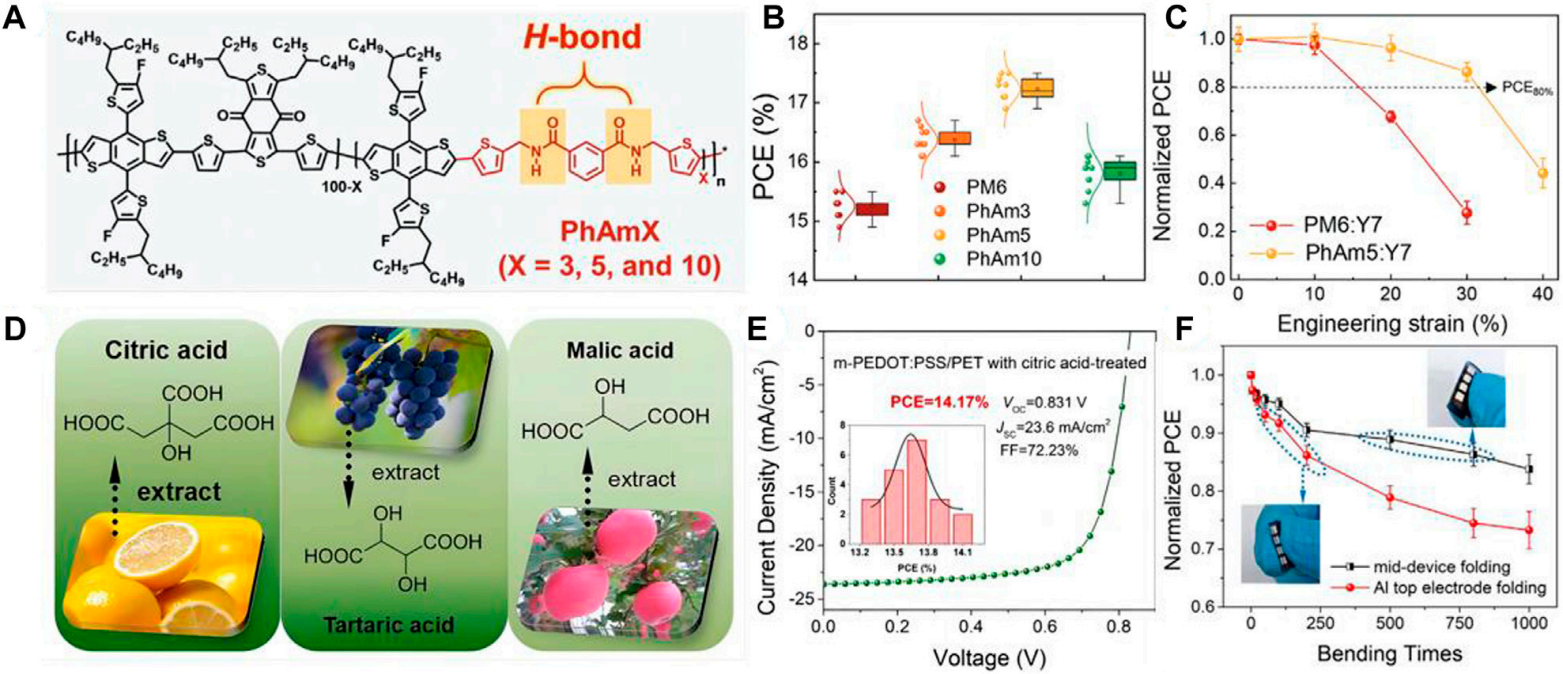
**(A)** Chemical structures of PM6 and PhAmX P_D_s. Adapted with permission from (Lee et al., 2022b); **(B)** Photovoltaic performances and related analyses of the Gaussian-fitted PCE distributions of PD:SMA blends. Adapted with permission from (Lee et al., 2022b); **(C)** Plots of normalized PCE *versus* engineering strain of the IS-OSC devices. Adapted with permission from (Lee et al., 2022b); **(D)** Molecular structures of citric acid, tartaric acid, and malic acid, respectively. Adapted with permission from (Xia et al., 2012); **(E)** Optimal J–V characteristics of flexible OSCs fabricated on m-PEDOT:PSS/PET with citric acid treatments (insert is corresponding histogram distribution of PCE counts for 20 individual devices). Adapted with permission from (Xia et al., 2012); **(F)** Changes in normalized PCEs of optimal flexible device based on citric-acid-treated m-PEDOT:PSS/PET electrode with mid-device and Al top electrode folding. Adapted with permission from (Xia et al., 2012).

Kim et al. developed a series of new polymer donors (P_D_s, PhAmX) featuring phenyl amide (N^1^, N^3^-bis((5-bromothiophen-2-yl)methyl) isophthalamide, PhAm)-based flexible spacer (FS) (Figure 1A) (Lee et al., 2022b). The PhAmX have different hydrogen bonding densities to pursue appropriate intermolecular hydrogen bonding interaction, which both have great charge mobility and excellent stretchability. Among them, the IS-OSCs based on the PhAm5 reached a high power conversion efficiency (PCE) of 12.73% (Figure 1B). Significantly, the PhAm5:Y7-based IS-OSC maintained over 90% of the initial PCE at 20% strain, which is much higher than the frequently-used PM6:Y7-based IS-OSC (68% of the initial PCE at 20% strain) (Figure 1C). In addition, the performance of the device under cyclic stretching/releasing is crucial for the sustainability of the IS-OSC. The PhAm5-based device maintained 86% of the initial PCE after 120 times stretching, non-etheless the PM6-based device showed only 41% of the initial PCE after the same number of cycles. In the PhAm5 system, although the π-conjugated system of the chain is destroyed by the introduction of the acid amides units, the hydrogen bonding induces the reduction of the intermolecular distances and makes better accumulation. The hydrogen bonding compensates for efficiency degradation due to the destruction of the π-conjugated system. Thus, the high durability and excellent performance under cyclic stretching of the hydrogen-bonding-based IS-OSC show high potential as the powerful source for soft-electronic devices in practical applications.

Except for the active layer, developing the highly stretchable transparent electrode material is also inevitable. Nevertheless, conventional OSCs usually utilized indium tin oxide (ITO) as the transparent electrode material, which with a high price and high rigidity (Xia et al., 2012). These factors have limited the practical applications of ITO on soft-electron devices. Thus, designing stretchable, foldable, and transparent electrodes also is meaningful for the development of wearable soft-electron devices. Recently, scientists have utilized a number of emerging flexible transparent electrodes to replace the ITO, including graphene, ultrathin metal, conducting polymers, etc. Among these materials, poly (3,4-ethylene dioxythiophene):poly (styrene sulfonate) (PEDOT:PSS) has been broadly used in flexible transparent electrodes, which posses well film-forming properties, high grade of transparency, and low cost (Chen et al., 2019). In addition, by doping with some acids in PEDOT:PSS films are confirmed as an effective strategy to enhance the performance of the devices. Wei Song et al. have designed the flexible OSCs by using the PEDOT:PSS (m-PEDOT:PSS)/polyethylene terephthalate (PET) films as the flexible transparent electrodes, which are doped with the eco-friendly acids, including citric acid, malic acid, and tartaric acid (Figure 1D) (Song et al., 2020). The carbonyl of the PET and the hydroxyl the acid can form hydrogen bonding in the system. Hydrogen bonding can significantly improve interfacial adhesion and reduce interface mechanical wear. Moreover, the deformation resistance and the environmental sustainability of the stretchable OSCs are obviously enhanced, which is caused by the intermolecular hydrogen bonding interaction. Thus, the green-acid-treated folding-flexible OSCs showed a high performance (PCE of 14.17%, V_OC_ of 0.831 V, J_SC_ of 23.60 mA cm^2^, and FF of 72.23%) with excellent stretchability (Figures 1E,F). This work is great potential for further implementation of stretchable, low-cost, and eco-friendly OSCs.

## 3 OFETs

Organic field-effect transistor (OFET) is an active device that uses an electric field to control the conductivity of solid organic materials. In electron devices, the OFETs are indispensable, which can transmit signals and control the operation of the devices (Fusco et al., 2022). Through molecular design and aggregate structural engineering, scientists have developed many high-charge mobility conjugated polymers for OFETs. With the development of soft-electron devices, designing corresponding stretchable OFETs are inevitable. Recently, many high-charge mobility with high stretchable OFETs have been successfully developed by hydrogen bonding designing strategies (Zhang et al., 2018), which may meet the requirement of emerging flexible electronics such as electronic skin, flexible displaying, etc. further improving charge mobility and promoting conjugated polymers to the flexible device are running in the fast lane (Wang et al., 2018a; Tien et al., 2021).

**FIGURE 2:**
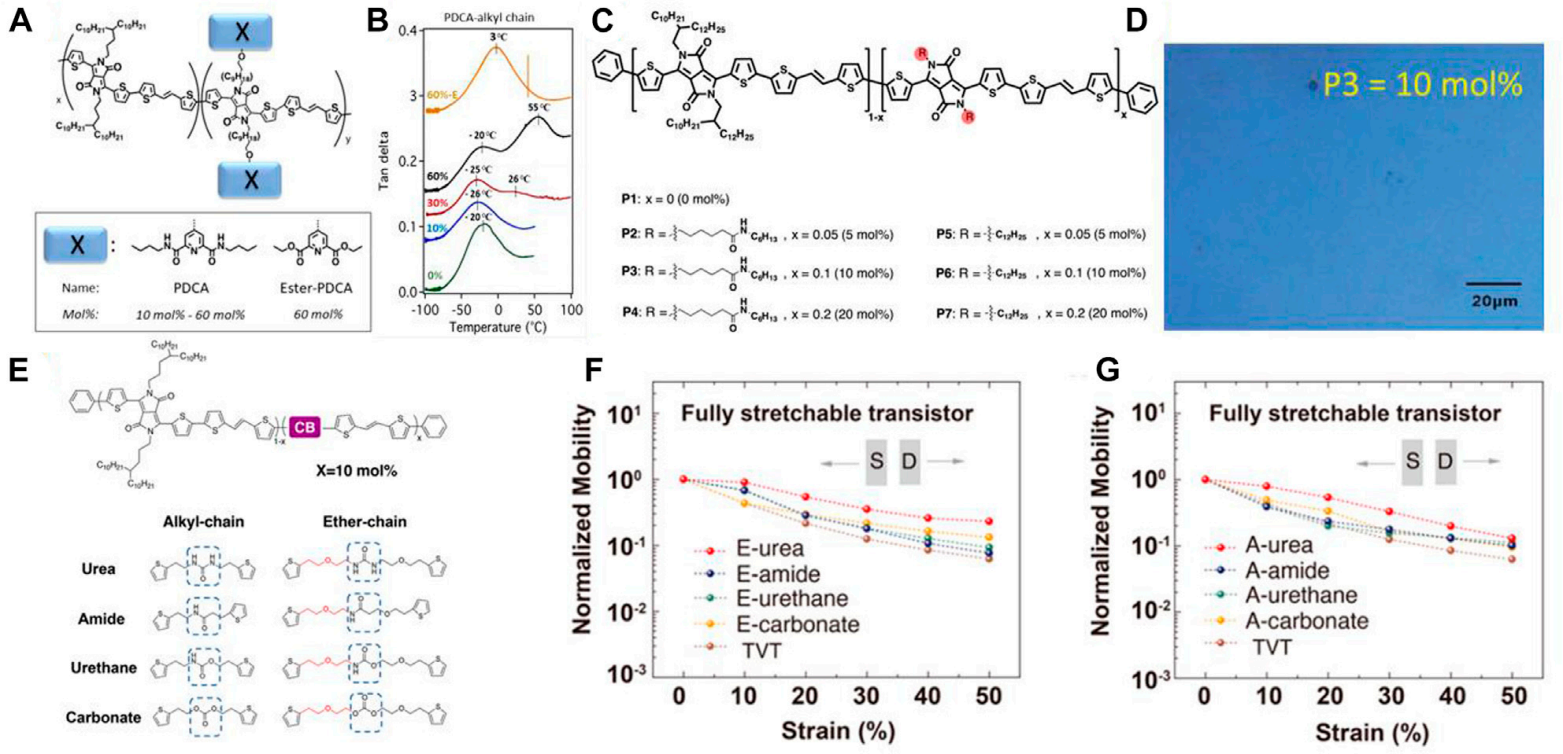
**(A)** The chemical structure of the DPP-based PDCA-alkyl side chain polymers. Adapted with permission from (Gasperini et al., 2019); **(B)** Dynamic mechanical analysis traces (tan δ curves) for semiconducting polymers bearing H-bonding units in the alkyl chains at different wt% of PDCA. Adapted with permission from (Gasperini et al., 2019); **(C)** Chemical structure of DPP-based conjugated polymers P1 to P7, incorporating up to 20mol% of amide-containing alkyl side chains and 20mol% of dodecyl side chains. Adapted with permission from (Ocheje et al., 2018); **(D)** optical microscopy images of P3 at 50% strain elongation. The scale bar is 20 μm. Adapted with permission from (Ocheje et al., 2018); **(E)** Chemical structure of the DPP-based conjugated polymer backbone and hydrogen bonding units. Adapted with permission from (Zheng et al., 2020); E-carbonate polymers during stretching in the fully stretchable transistor configuration, with charge transport parallel to the strain direction. Average mobility of different H-bonding conjugated polymers during stretching in the fully stretchable transistor configuration (normalized by the mobility at 0% strain), with charge transport parallel to the strain direction: **(F)** ether-chain and **(G)** alkyl-chain. All mobility values are averaged and extracted from at least six devices. Adapted with permission from (Zheng et al., 2020).

Incorporating side chains into the polymer is a mature design strategy to enhance the performance of OFETs. For example, Bao’s group introduced hydrogen bonding units PDCA (2,6-pyridine carboxamide) into Diketopyrrolopyrrole (DPP)-based donor−acceptor conjugated polymer OFETs (Figure 2A) (Gasperini et al., 2019). Compared with commonly DPP-based OFETs, the hydrogen bonding interaction reduced the intermolecular distance (from 24.02 to 22.87 Å) and induced better π-π accumulation. They found that the hydrogen bonding is more sturdy than the backbone in the hydrogen bonding system (Figure 2B). That means the hydrogen bonding can buffer the effect while the materials are strained, which can significantly improve the stretchability and resistance to strain. Samely, Ocheje’s group incorporated amide-containing alkyl chains in DPP-based conjugated polymers as stretchable OFETs (Figure 2C) (Ocheje et al., 2018). The resulting polymers with 10% hydrogen bonding units showed a maximum stretchability of 75% elongation (Figure 2D), which maintains a high retention rate of the morphology under the tensile state. Non-etheless, the hydrogen bonding side chains also can be extended to other polymer backbone structures to develop various high-performance OFETs.

Besides introducing hydrogen bonding side chains in the OFETs system, developing different hydrogen bonding densities in the backbone also is a popular research direction (Oh et al., 2016). The hydrogen bonding is able to induce better aggregation and crystallinity in as-casted thin films, which can result in a higher modulus and crack on-set strain. This property is highly correlated with the density and strength of hydrogen bonding. On this basis, Bao’s group designed various diketopyrrolopyrrole (DPP)-based conjugated polymer backbones with various densities and intensities of hydrogen bonding units, and systematically investigate the effects of hydrogen bonding interactions on the performance of OFETs (Figure 2E) (Zheng et al., 2020). They found that the hydrogen bonding self-association constant >0.7 (the Urea) can significantly improve the resistance to deformation and crack on-set strain. Additionally, introducing the ether chain contributes to better hydrogen bonding interaction and better electrical performance under strain. Then they fabricated the stretchable OFETs to evaluate their electrical performances under the strain states. The result shows that the hydrogen bonding induced better crystallization and improved the modulus of the system (Figures 2F,G). Nevertheless, the higher modulus leads to better charge mobility. Thus, regulating the densities and intensities of hydrogen bonding in the backbone can provide guidelines for designing various stretchable OFETs.

## 4 Conclusions and outlook

Hydrogen bonding-induced organic semiconductors have many advantages in stretchable electron devices, including designability, low cost, stretchability, etc. Moreover, through different design strategies, we can develop various functional stretchable organic semiconductors as we want. The development of stretchable organic semiconductors could promote the development of wearable soft-electron devices. On one hand, stretchable organic semiconductors can provide power for soft-electron devices such as stretchable OSCs. On the other hand, designing various stretchable organic semiconductors let the development of soft-electron devices quicker and more diversified.

Although hydrogen bonding-induced stretchable organic semiconductors have developed for many years, this technology is still in its infancy. For further development of soft-electron devices, there are still some problems that need to be solved. (i) At present, many hydrogen bonding-induced stretchable organic semiconductors lack deep-level research. (ii) Developing more practical applications, such as artificial skin, wearable electronic devices, electronic wallpaper, etc. (iii) Developing different types of hydrogen bonding-induced organic semiconductors, such as chemical hydrogen bonding, physical hydrogen bonding and dielectric hydrogen bonding. (iv) Designing Hydrogen bonding-induced organic semiconductors with multiple advantages, including sustainability, stretchability, charge mobility, foldability, etc.
